# Expression Patterns of the Neuropeptide Urocortin 3 and Its Receptor CRFR2 in the Mouse Central Auditory System

**DOI:** 10.3389/fncir.2021.747472

**Published:** 2021-11-12

**Authors:** Sara Pagella, Jan M. Deussing, Conny Kopp-Scheinpflug

**Affiliations:** ^1^Division of Neurobiology, Faculty of Biology, Ludwig-Maximilians-University Munich, Munich, Germany; ^2^Research Group Molecular Neurogenetics, Max Planck Institute of Psychiatry, Munich, Germany

**Keywords:** urocortin, CRFR2, auditory, stress signaling, multimodal, volume transmission, calyx of Held synapse

## Abstract

Sensory systems have to be malleable to context-dependent modulations occurring over different time scales, in order to serve their evolutionary function of informing about the external world while also eliciting survival-promoting behaviors. Stress is a major context-dependent signal that can have fast and delayed effects on sensory systems, especially on the auditory system. Urocortin 3 (UCN3) is a member of the corticotropin-releasing factor family. As a neuropeptide, UCN3 regulates synaptic activity much faster than the classic steroid hormones of the hypothalamic-pituitary-adrenal axis. Moreover, due to the lack of synaptic re-uptake mechanisms, UCN3 can have more long-lasting and far-reaching effects. To date, a modest number of studies have reported the presence of UCN3 or its receptor CRFR2 in the auditory system, particularly in the cochlea and the superior olivary complex, and have highlighted the importance of this stress neuropeptide for protecting auditory function. However, a comprehensive map of all neurons synthesizing UCN3 or CRFR2 within the auditory pathway is lacking. Here, we utilize two reporter mouse lines to elucidate the expression patterns of UCN3 and CRFR2 in the auditory system. Additional immunolabelling enables further characterization of the neurons that synthesize UCN3 or CRFR2. Surprisingly, our results indicate that within the auditory system, UCN3 is expressed predominantly in principal cells, whereas CRFR2 expression is strongest in non-principal, presumably multisensory, cell types. Based on the presence or absence of overlap between UCN3 and CRFR2 labeling, our data suggest unusual modes of neuromodulation by UCN3, involving volume transmission and autocrine signaling.

## Introduction

Temporary changes in hearing during stressful situations or episodes of anxiety or sadness are commonly experienced by humans and animals (Neuser and Knoop, 1986; Schmitt et al., 2000; Horner, 2003; Kadner et al., 2006; Mazurek et al., 2010; Pacheco-Unguetti and Parmentier, 2014; Lin et al., 2016; Szczepek et al., 2018). Such changes can range from decreased attention to sincere auditory hallucinations (Hoskin et al., 2014). More profound and chronic stress system dysregulation such as with post-traumatic stress disorder (PTSD) can come with a plethora of associated auditory abnormalities (Shalev et al., 2000; Guthrie and Bryant, 2005; Karl et al., 2006; Clifford et al., 2018). Conversely, specific acoustic qualities of sound can trigger stress-like sensations or even fear in both humans and animals, with the acoustic startle response being the most prominent example (Davis, 1984; Trost et al., 2012; Koelsch et al., 2013; Koelsch, 2014; Behler and Uppenkamp, 2020; Hegewald et al., 2020).

There is a clear reciprocity between stress and sensory systems. On one hand, the stress response relies on incoming information from the external environment provided by sensory systems to execute its function of maintaining allostasis, while on the other hand, sensory systems have to adapt constantly to changes in external conditions that are potentially threatening to the animal’s current state. Since ears, unlike eyes, are open and sensing for 24 h a day, the auditory system plays a pivotal role in survival and has to be protected not only from noise trauma, but also from degenerative damage resulting from non-auditory stressors such as infections and head trauma. Hence, strong two-way interactions between stress signaling and the auditory system are warranted.

In the auditory system, the medial subdivision of the medial geniculate body (MGBm), the external cortex of the inferior colliculus (ICe) and the auditory cortex (Au) are well-known areas of connection to stress pathways. In particular, the MGBm-amygdala-pathway is known to be involved in auditory fear conditioning and in attributing emotional salience to sounds (LeDoux et al., 1984). The ICe receives hypothalamic input (Sakanaka et al., 1987) which might be involved in circadian regulation of stress, since both areas contain independent clocks (Park et al., 2016). The primary auditory cortex (Au1) sends direct projections to the lateral amygdala, which in turn projects to the auditory association cortex (Romanski and LeDoux, 1993; McDonald, 1998; Phelps and LeDoux, 2005). On top of these discrete connections, a wide network of mostly serotoninergic and cholinergic projections coming from the reticular formation innervate the auditory system throughout its extent from cochlea to cortex (Klepper and Herbert, 1991; Hurley and Hall, 2011; Schofield et al., 2011). Noradrenergic inputs, which mostly target the dorsal (DCN) and the granule cell domain (GCD) of the cochlear nucleus (CN), the ICe, the superior olivary complex (SOC), and the Au are even less understood (Levitt and Moore, 1978; Klepper and Herbert, 1991; Mulders and Robertson, 2001).

Non-auditory areas are crucial in shaping auditory responses by stressors coming from other sensory modalities. For example, olfactory stimulation with predator odor has been shown to trigger changes in neuronal firing rates of the locus coeruleus, a brainstem nucleus that receives auditory input and releases norepinephrine (Curtis et al., 2012). There, presentation of a stressor such as corticotropin-releasing factor (CRF) or a predator odor increases spontaneous tonic firing and decreases sound-evoked phasic firing of the neurons. Such a shift from tonic to phasic firing in locus coeruleus neurons is suggested to facilitate different behavioral reactions (Aston-Jones and Cohen, 2005).

The activation of the hypothalamic-pituitary-adrenal axis (HPA) is commonly regarded as the cornerstone of the stress response. Briefly, this entails release of CRF from hypothalamic paraventricular nucleus’ neurons into the anterior pituitary gland, which in turn secretes adrenocorticotropic hormone (ACTH) that, upon reaching the adrenal glands, stimulates cortisol synthesis and release. Cortisol targets specific cell groups throughout the body *via* blood circulation (Charmandari et al., 2005). In its target cells, cortisol binds to its nuclear receptor and elicits the transcriptional modifications to adapt cellular function. It takes about 30 min to reach sufficient ACTH concentrations in the blood for cortisol to be synthetized (Sapolsky et al., 2000), a delay that could impede the timeliness of the response.

Thanks to the pioneering work of the Vetter-lab, a much faster, HPA axis-independent stress axis has been discovered in the cochlea, that not only involves all the constituents for cortisol production, but also a local CRF system (Basappa et al., 2012). The CRF system consists of two receptors CRFR1 and CRFR2, four ligands and the non-membrane-bound CRF-binding-protein. The four ligands, CRF, urocortin 1 (UCN1), 2 (UCN2), and 3 (UCN3) display different affinities for the two receptor types. CRF and UCN1 are most affine to CRFR1 but may also at bind to CRFR2 at very high concentrations (Chang et al., 1993; Chen et al., 1993; Vita et al., 1993; Perrin et al., 1995; Deussing and Chen, 2018). In contrast, UCN2 and UCN3 bind exclusively to CRFR2 (Lewis et al., 2001; Reyes et al., 2001; Deussing et al., 2010).

The most abundant stress peptide in the auditory system is CRF, which is expressed in the cochlea (Basappa et al., 2012), the principal neurons of the lateral superior olive (LSO), the ventral nucleus of the lateral lemniscus (VNLL), the inferior colliculus (IC), and the medial geniculate body (MGB) (including the peripeduncular and posterior intralaminar nuclei), the Brodman areas 20, 39, 40, and 41 and, although much weaker, in the deep layers of the DCN and lateral part of the medial nucleus of the trapezoid body (MNTB) (Imaki et al., 1991). UCN1 expression in the auditory system is much more distinct and has been reported only in a small subset of lateral olivocochlear bundle cells (LOC) with high characteristic frequencies (CF) as well as in the neuropil of the DCN deep layers and IC (Kozicz et al., 1998; Kaiser et al., 2011).

To date, no data are available that suggest UCN2 expression in the auditory system (Lewis et al., 2001). That makes UCN3 the primary ligand for CRFR2. So far, UCN3 expression in the auditory system was reported in the cochlea and SOC (Lewis et al., 2001; Li et al., 2002; Fischl et al., 2019). However, the physiological importance of UCN3 and its receptor CRFR2 for auditory function has been emphasized by knockout models of UCN3 and CRFR2, both showing enhanced vulnerability to noise trauma (Graham et al., 2010; Fischl et al., 2019). Although noise trauma and systemic stress are two major causes of hearing loss in humans (Masuda et al., 2012), our knowledge of the UCN3–CRFR2 contribution to auditory signal processing is rather limited.

In the present study, we take advantage of two reporter mouse lines, one for UCN3 and the other for its receptor CRFR2 to provide an extensive description of UCN3 and CRFR2 expression in the central auditory system. Immunocytochemistry was used for co-labeling the neurons highlighted by the reporter to allow the best possible identification of specific areas and cell types.

Characteristic expression patterns throughout this study showed UCN3 expression in principal auditory neurons and CRFR2 expression in non-principal/multimodal neurons of the same nucleus. Fewer areas revealed neurons that express both the ligand as well as the receptor. Together, both types of expression suggest that volume transmission as well as autocrine regulation are possible signaling mechanisms for UCN3.

## Materials and Methods

All experimental procedures were reviewed and approved by the Bavarian district government (ROB-55.2-2532.Vet_02-18-1183) and were done according to the European Communities Council Directive (2010/63/EU). Mice were housed in a vivarium with a normal light–dark cycle (12 h light/12 h dark) and food and water *ad libitum*.

### Mouse Models

Experiments were conducted on four UCN3 reporter mice (UCN3 tdTom) and three CRFR2 reporter mice (CRFR2 tdTom) of both sexes (five males and two females). After weaning, mice were separated by sex and group housed with same sex littermates until used in the experiment. In this absence of deliberate stressors, no differences in expression patterns were observed between males and females.

Reporter mice were generated by breeding UCN3-Cre mice [Tg(UCN3-Cre)KF31Gsat; The Gene Expression Nervous System Atlas (GENSAT) Project; Mutant Mouse Resource & Research Centers (MMRC) stock no: 033033-UCD] or CRFR2-Cre mice (Henckens et al., 2017) with R26^CAG–LSL–tdTomato^ mice (Ai9, The Jackson Laboratory, Bar Harbor, ME, United States; stock no: 007905) as previously described (Shemesh et al., 2016).

### Immunohistochemistry

Mice received an overdose of pentobarbital (400 mg kg^–1^ body weight; I.P.) and were perfusion-fixed with 4% paraformaldehyde (PFA) intracardially. Following overnight postfixation in 4% PFA, brainstems were sectioned coronally at 50 μm using a vibrating blade microtome (V1200S, Leica, Wetzlar, Germany). After rinsing in phosphate-buffered saline (PBS), sections were transferred to a blocking solution containing 1% bovine serum albumin, 0.5% Triton X-100, and 0.1% saponin in PBS. The floating sections were then incubated for 48 h at 4°C in blocking solution containing primary antibodies, which were used in different combinations as specified in the figures. Sections were washed three times in PBS for 15 min and were then incubated with secondary antibodies overnight at 4°C. Antibodies against calbindin D28K (SWANT, #07F Burgdorf, Switzerland, 1:300) were combined with secondary antibodies Alexa 488 (Dianova anti-rabbit, #115-545-206, Hamburg, Germany, 1:200). Antibodies against parvalbumin PV-28 (SWANT, Burgdorf, Switzerland, 1:500) were combined with secondary antibodies AMCA (Dianova, anti-mouse, Hamburg, Germany, #715-156-150, 1:100). Antibodies against VGluT1 (Synaptic Systems #135304, Göttingen, Germany, 1:500) were combined with secondary antibodies Alexa 647 (Dianova, anti-guinea pig, Hamburg, Germany, #706-605-148). Antibodies against vesicular glutamate transporter type 2 (VGluT2) (Synaptic Systems, Göttingen, Germany, #135402, 1:500) were combined with secondary antibodies Alexa 488 (Dianova anti-rabbit, #115-545-206, Hamburg, Germany, 1:200). Antibodies against glycine transporter type 2 (GlyT2; Millipore #1773, 1:1000) were combined with secondary antibodies Alexa 647 (Dianova, anti-guinea pig, Hamburg, Germany, #706-605-148). Antibodies against VChat (Synaptic System #139105, Göttingen, Germany, 1:200) were combined with secondary antibodies Alexa 647 (Dianova, anti-guinea pig, Hamburg, Germany, #706-605-148). Sections were washed in PBS, mounted on slides and coverslipped with vectashield mounting medium (Vector Laboratories, Burlingame, CA, United States).

### Confocal Microscopy and Image Analysis

Confocal optical sections were acquired with a confocal laser-scanning microscope equipped with HCX PL APO CS 20×/NA0.7 and HCX PL APO Lambda Blue 63×/NA1.4 immersion oil objectives (Leica). Fluorochromes were visualized with excitation wavelengths of 405 nm (emission filter 410–430 nm) for amino-methylcoumarin (AMCA), 488 nm (emission filter 510–540 nm) for Alexa 488, 561 nm (emission filter 565–585 nm) for Cy3, and 647 nm (emission filter 663–738 nm) for Alexa 647. For each optical section, the images were collected sequentially for the different fluorochromes. Stacks of 8-bit grayscale images were obtained with axial distances of 290 nm between optical sections and pixel sizes of 120–1520 nm depending on the selected zoom factor and objective. To improve the signal-to-noise ratio, images were averaged from three successive scans. RGB stacks, montages of RGB optical sections and maximum-intensity projections were assembled using the ImageJ StackGroom plugin. Color schemes were adjusted to C-M-Y-W.

## Results

Expression patterns of UCN3 and CRFR2 in the cochlea and spiral ganglion neurons were reported in detail before (Graham et al., 2010; Fischl et al., 2019), so that here, we start with the description of UCN3 and CRFR2 expression in the CN. From there we proceed to the SOC, the LL, the IC, and the MGB. The auditory cortex exhibited hardly any UCN3 or CRFR2 expression so that we focused on subcortical auditory areas.

### Cochlear Nucleus

**FIGURE 1:**
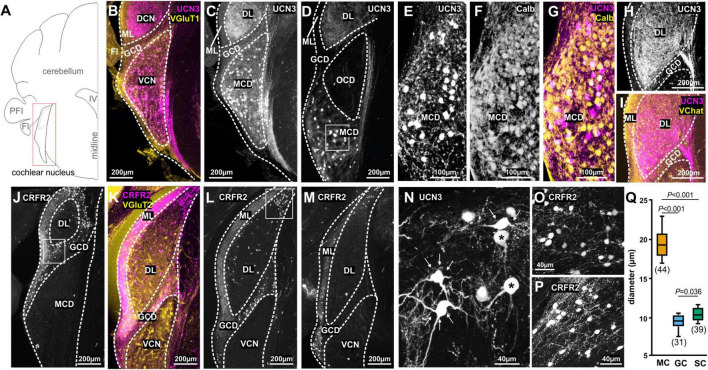
Spatial segregation of UCN3 and CRFR2 expression in the cochlear nucleus. **(A)** Schematic coronal section showing the cochlear nucleus as the region of interest. PFI, paraflocculus; FI, flocculus; IV, fourth ventricle. **(B)** UCN3 expression (magenta) in the VCN and in the DCN VGluT1 (yellow) labeling dominates in the molecular layer (ML) of the DCN and the granule cell domain (GDC). **(C)** Same image as in **(B)**. UCN-expression in neuronal cell bodies of the VCN magnocellular domain (MCD) and in dense fiber networks in the deep layer (DL) of DCN. **(D)** More caudal coronal section highlighting the lack of UCN3 expression in the octopus cell domain (OCD). Square in the MCD indicates UCN3-positive cell bodies in the MCD, shown in higher magnification in **(M)**. **(E–H)** Co-staining of UCN3-positive neurons in the MCD **(E)** with calbindin **(F)**. Neurons co-labeled for UCN3 (magenta) and calbindin (yellow) appear white. **(H,I)** UCN3-expressing fibers in the DCN deep layer (DL) do not enter the molecular layer (ML), which is characterized by yellow VChat labeling in **(I)**. **(J–L)** Coronal sections in **(J–L)** show CRFR2 expression in different rostro-caudal positions along the cochlear nucleus. **(J)** CRFR2 expression in the parallel fibers of the DCN molecular layer (ML), in granule cells of the granule cell domain (GCD), but not in the MCD of the VCN. Square in the GCD indicates UCN3-positive cell bodies shown in higher magnification in **(O)**. **(K)** Co-staining of CRFR2 (magenta) and VGluT2 (yellow) in a more caudal image compared to **(J)**. White color in the GCD and the ML suggest co-labeling. **(L)** Same image as in **(K)**. CRFR2 expression in ML parallel fibers, in GCD granule cells and in neurons of the small cell cap (white square), which are shown in higher magnification in **(P)**. **(M)** CRFR2 expression in a more caudal section compared to **(J,K)**, depicting again parallel fibers, granule cells, and small cells. **(N)** Higher magnification of UCN3-positive MCD neurons reveal a globular (asterisk) or multipolar shape (white arrows). **(O)** Higher magnification of CRFR2-expressing granule cells. **(P)** Higher magnification of CRFR2-expressing small cap cells. **(Q)** Quantification of cell diameters of magnocellular cells of the VCN (MC), granule cells (GC), and small cells (SC). ANOVA was used to test for statistical differences.

The mouse CN (Figure 1A) is divided into four main areas: anteroventral (AVCN), posteroventral (PVCN), and dorsal (DCN) CN as well as the granule cell domain (GCD) (Harrison and Irving, 1965; Mugnaini et al., 1980; Willard and Ryugo, 1983). AVCN and PVCN contain similar neuronal cell types such as globular (GBCs) and spherical bushy cells (SBCs), stellate cells (T-stellate and D-stellate), and little cells (LCs), all of which are regarded as principal auditory neurons (Cant, 1993; Oertel et al., 2011; Ngodup et al., 2020). In addition, the PVCN also contains octopus cells (OCs) which inhabit their own domain near the auditory nerve root region (Oertel et al., 2000). Location, morphology and co-labeling with recognized markers were used to identify these neurons.

The UCN3 tdTom reporter mouse showed an abundance of UCN3-positive neuronal cell bodies in the magnocellular domain (MCD) of AVCN and PVCN (Figures 1B–G). Auditory nerve terminals innervate the VCN tonotopically, with ventro-lateral neurons receiving inputs originating from the apex of the cochlea and therefore being responsive to low-frequency sounds. Neurons located more dorso-medially receive input from the base of the cochlea, which is sensitive to higher sound frequencies (Liberman, 1991, 1993). UCN3-positive neurons are found predominantly in the ventro-lateral, low-frequency part of the AVCN and progress to occupy more intermediate areas in the PVCN. No UCN3 expression was observed in the octopus cell domain (OCD), which is visualized by the empty space visible in Figure 1D. Only some of the UCN3-positive neurons in the MCD of the VCN co-localize with the Ca^2+^ binding protein calbindin (Figures 1E–G). Therefore we used size measures to assess if these UCN3-positive neurons qualify as canonical principal auditory neurons, which in the MCD are quite large in diameter (Oertel et al., 2000; Bazwinsky et al., 2008; Lauer et al., 2013). In addition to the large soma size (mean ± SD: 19.59 ± 2.17 μm; *n* = 44 neurons; 3 mice; Figures 1N,Q), morphological characteristics such as a round soma, typical bush-like dendrites, and projections to the contralateral MNTB and ipsilateral LSO allowed us to identify some UCN3-positive neurons as bushy cells (Figure 1N; Webster and Trune, 1982). However, a large number of UCN3-positive neurons in the VCN seem to be stellate cells based on their distinct multipolar (stellate) shape (Figure 1N) and by prominent ascending fibers originating from these cells and profusely innervating the ipsilateral DCN (Figures 1H,I). The observation of many UCN3-positive axons leaving the VCN, crossing in the trapezoid body, but not terminating in a calyx of Held suggest a T-stellate cell origin, as the “T” in T-stellate was, indeed, given to underline the fact that very often these neurons send axons across to the other side through the trapezoid (tz) body (Oertel et al., 1990). Other UCN3-positive stellate cell axons connect the VCN to the DCN. These fibers could be of D- or T-stellate cell origin and the neuronal somata of these cell types are found rostrocaudally in the VCN. The innervation pattern of this bundle is similar to that of metabotropic acetylcholine receptor type 2 (AChR M2) recently described (Malfatti et al., 2021). Using an AChR M2 reporter mouse, this bundle was interpreted as originating from VCN T-stellate cells. After entering the DCN, the UCN3-positive fibers span the whole of the deep layers, but without entering the molecular domain, which is characterized by an abundance of cholinergic inputs (Figures 1G,H; Fujino and Oertel, 2001). In conclusion, the UCN3 tdTom reporter shows that in the VCN, a small number of globular bushy cells and a larger number of stellate cells express UCN3. Both, bushy cells and T-stellate cells are found in the MCD of the nucleus and are involved in the faithful transmission of sound information from the cochlear nerve. In contrast to the MCD, the granular cell domain (GCD) does not exhibit any UCN3-positive cells or fibers (Figures 1C,D). Instead, UCN3-positive projections originating from the VCN innervate large parts of the DCN, spanning the deep (polymorphic) and the fusiform layers up to the molecular layer but not trespassing into the GCD nor the molecular layer. The GCD forms a more or less defined area between the magnocellular core of the VCN and the DCN (Mugnaini et al., 1980). The GCD consists mainly of granule cells as well as some other less frequent cell types (unipolar brush cells, chestnut cells, and Golgi cells) (Floris et al., 1994; Weedman et al., 1996; Yaeger and Trussell, 2015). Cells in the GCD are considered non-principal neurons. Although they respond to sound stimulation, especially at high intensities, they mainly integrate sound information with multisensory inputs, rather than to encode straightforward sound properties (Yang et al., 2005; Flores et al., 2015). Much of this multisensory, integrative processing takes place in the molecular layer. The axons of the granule cells populate the molecular layer of the DCN as parallel fibers where they interact with the dendrites of fusiform cells and cartwheel cells. Inputs from non-auditory areas such as the pontine nuclei, the nucleus cuneatus, the vestibular nucleus or the spinal trigeminal nucleus (Zhao et al., 1995; Wright and Ryugo, 1996; Ohlrogge et al., 2001; Ryugo et al., 2003; Zhou and Shore, 2004; Zhan et al., 2006; Zhan and Ryugo, 2007) tend to be positive for the VGluT2, whereas primary auditory inputs are predominantly VGluT1 positive (Zhan and Ryugo, 2007; Zeng et al., 2011).

The expression pattern of CRFR2, differs considerably from the UCN3 expression patterns. In the CN, CRFR2 is expressed almost exclusively in neurons and axonal tracts of the GCD (Figures 1J–M). Cell bodies and axons span the entire rostrocaudal extent of the GCD, including the lamina between VCN and DCN (Figures 1J–M). The morphology of these cells suggests them to be granule cells (Figure 1O). They exhibit the characteristic small size with a mean (±SD) diameter of 9.51 ± 1.02 μm (*n* = 31 neurons; 3 mice; Figure 1Q). This size is significantly smaller then neurons of the MCD (ANOVA: *p* ≤ 0.001; Figures 1O,Q). Another prominent feature of granule cells are their axons, which take the characteristic parallel course with respect to the nucleus borders. The CRFR2-positive fibers inhabit the DCN molecular layer, which is also VGluT2-positive and predestines them as granule cells’ parallel fibers (Figures 1J–M; Rubio et al., 2008). In addition to CRFR2 labeling of granule cells and parallel fibers, another population of slightly bigger CRFR2-positive cells were observed at the dorsolateral edge of the DCN (Figures 1J–L,P). These neurons are slightly larger in diameter (mean ± SD: 10.45 ± 0.88 μm; *n* = 39 neurons; 3 mice) than granule cells (ANOVA: *p* = 0.036; Figures 1O–Q), but clearly smaller than magnocellular cells (ANOVA: *p* ≤ 0.001; Figures 1N,Q). Their size and the fact that they are not entirely embedded in the parallel fiber mesh, but rather somewhat underneath suggests them to be small cap cells (Osen, 1969; Cant, 1993; Ryugo, 2008). Small cap cells receive input from medial olivocochlear complex (MOC) neurons in the ventral nucleus of the trapezoid body (VNTB) and project back to the VNTB and also to the MGB (Benson and Brown, 1990; Ryan et al., 1990; Brown et al., 1991; Thompson and Thompson, 1991; Ye et al., 2000; Malmierca et al., 2002; de Venecia et al., 2005; Darrow et al., 2012; Hockley et al., 2021). Both areas, the GCD and the small cap cell location contain CRFR2 positive fibers. In contrast to the UCN3 expression, only a few cells in the magnocellular cores of DCN and VCN were CRFR2 positive. CRFR2-positive cells in in the AVCN are most likely GBCs that form calyces of Held in the lateral part of the MNTB as we will describe in the next paragraph.

### Superior Olivary Complex

The mouse SOC is a cluster of interacting nuclei serving essential functions of auditory processing which require both temporal precision and binaural integration. Roughly, the SOC nuclei can be sorted into those involved in sound source localization in the horizontal plane like the medial and lateral nucleus of the trapezoid body (LNTB and MNTB) and the medial superior olive (MSO) and LSO and those that are not involved in sound localization (Grothe and Pecka, 2014). The latter include the periolivary nuclei like the VNTB, the superior paraolivary nucleus (SPN), and the dorsal periolivary nucleus (DPO), whose function seems to vary between species and ranges from efferent feedback to encoding communication sounds (Frank and Goodrich, 2018; Kopp-Scheinpflug et al., 2018).

**FIGURE 2:**
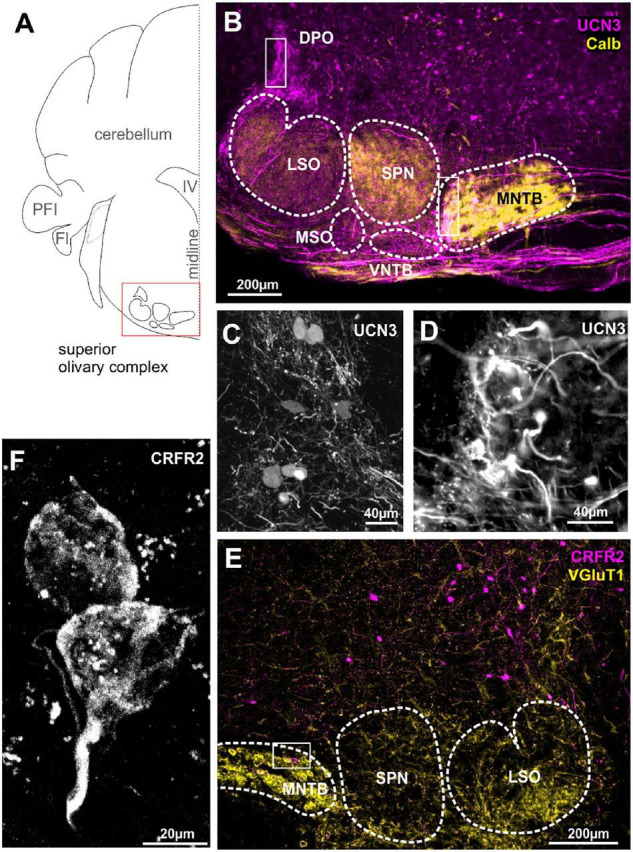
Urocortin 3-positive neurons and fibers dominate over sparse CRFR2 expression in the SOC. **(A)** Schematic coronal section showing the superior olivary complex (SOC) as the region of interest. PFI, paraflocculus; FI, flocculus; IV, fourth ventricle. **(B)** Dense UCN3-positive fiber bundles (magenta) enter the SOC from the contralateral side of the brain and innervate the MNTB, SPN, and LSO. Calbindin (yellow) is used as counter stain for SOC nuclei. UCN3-positive neuronal cell bodies are present in the VNTB and in the DPO (white square). Calyces of Held in the lateral, low-frequency MNTB are UCN3-positive (white square in MNTB). The data shown in **(A–C)** corroborate previously published findings (Fischl et al., 2019), but are shown here for direct comparison with the data on the UCN3 receptor CRFR2. **(C)** Higher magnification of UCN3-positive DPO neurons and fibers. **(D)** Higher magnification of UCN3-positive calyx synapses in the lateral MNTB. **(E)** Sparse CRFR2 expression (magenta) in the SOC. Instead, CRFR2-positive neurons are found in the reticular formation dorsal to the SOC. VGluT1 (yellow) is used a counterstain. **(F)** CRFR2 expression in the calyces of Held.

We previously reported the expression of UCN3 in the auditory brainstem nuclei with respect to the protection of the auditory system from sound over-exposure (Fischl et al., 2019). Surprisingly, this protective function was not accompanied by high numbers of UCN3-positive neuronal cell bodies in the SOC, but rather with an abundance of UCN3 expressing axons and synaptic terminals in this area (Figures 2A,B). A few UCN3-positive somata are found at the dorsomedial edge of the SPN, the VNTB and in a poorly defined area around the dorsolateral edge of LSO possibly corresponding to the DPO (Figures 2B,C). Cells in the VNTB and the DPO areas are part of the MOC system. Distinct UCN3 expression was observed at the calyces of Held (Figures 2B,D). This calyceal UCN3 expression was confined to the calyces contacting MNTB neurons in the lateral subdivision of the MNTB (Figure 2B). According to the tonotopic organization of the MNTB, the neurons that receive the UCN3-positive input are low-CF cells. The UCN3 expression in the calyces of Held corroborate the UCN3-positive AVCN neurons to be identified and GBCs (Felmy and Schneggenburger, 2004). Other, contralaterally originating, ascending fibers terminate in the MSO and VNTB area. In addition, we observed ipsilateral ascending fibers, which contact lateral, low-frequency LSO neurons. The origin of these UCN3-positive axons is most likely in the AVCN bushy cells. Another distinctive bundle of UCN3-expressing fibers terminates at the level of the DPO (Figures 2B,C). Overall, compared to the CN, UCN3 expression in the SOC neurons is scarce. This is reflected by equally sparse CRFR2 expression in the SOC. However, the expression of CRFR2 in lateral calyces of the MNTB (Figure 2F) as well as in fibers in the lateral limb of the LSO (Figure 2E) should be highlighted, because they mirror the UCN3 expression in these nuclei and suggest a possible autocrine regulation of CRFR2 (Figures 2B,E).

### Lateral Lemnisci

**FIGURE 3:**
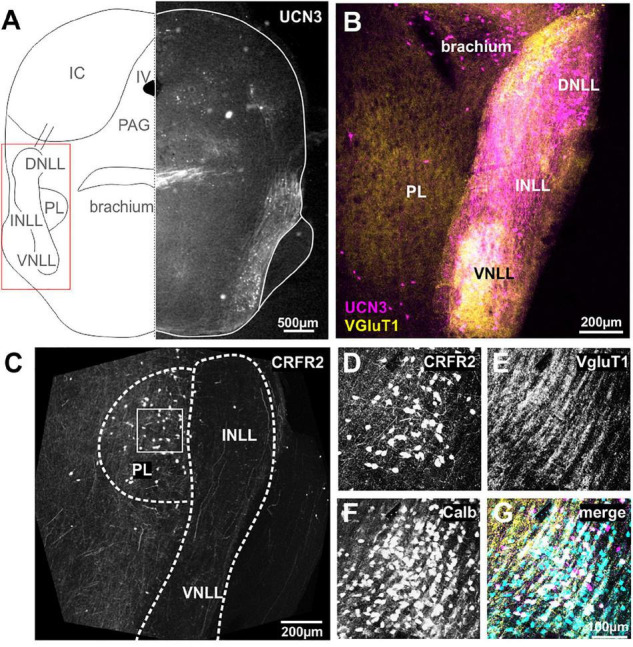
Neurons and fibers of the lateral lemniscus express UCN3 but not CRFR2. **(A)** Schematic coronal section showing the nuclei of the lateral lemniscus (NLL) as the region of interest. IV, fourth ventricle; PL, paralemniscal nucleus; PAG, periaqueductal gray; IC, inferior colliculus. Left side shows UCN3 expression in the brachium, the intermediate (INLL), and the ventral (VNLL) nuclei of the lateral lemniscus. **(B)** Higher magnification of the NLL shows UCN3 expression (magenta) in the brachium and all three NLLs. White color in the VNLL suggests co-labeling of UCN3 (magenta) and VGluT1 terminals. The paralemniscal (PL) areas are medial to the INLL are positive for VGluT1, but show no UCN3 expression. **(C)** Instead the PL shows strong CRFR2 labeling. **(D)** CRFR2-positive neural cell bodies in the PL. **(E)** Dense network of VGluT1-positive fibers surrounding PL neurons. **(F)** PL neurons are calbindin-positive. **(G)** Co-labeling of CRFR2 (magenta), VGluT1 (yellow), and calbindin (turquoise) suggests that a subpopulation of calbindin-positive neurons also express CRFR2 (white color).

The cochlear nuclei and the SOC connect to the IC *via* a fiber bundle that passes along the lateral edge of the brainstem and is termed lateral lemniscus (Figure 3A). Within these fibers, there are three distinct neuronal populations, the dorsal, the intermediate and the ventral nucleus of the lateral lemniscus, DNLL, INLL, and VNLL, respectively (Merchán et al., 1997; Oertel and Wickesberg, 2002; Ito et al., 2011). The nuclei of the LL receive their input predominantly from the contralateral VCN and the ipsilateral SOC (Glendenning et al., 1981). Additionally, the DNLL exchanges reciprocal projections with the contralateral DNLL (Oliver and Shneiderman, 1989). Although there is still much to learn about the LL’s role in auditory processing, a main function seems to be to send a fast feed-forward inhibition as well as a long-lasting inhibition into the IC (Ammer et al., 2015). The INLL receives additional input from the contralateral paralemniscal nucleus (Kelly et al., 2009). The nuclei of the lateral lemniscus (NLLs) seem to provide a major link between the auditory and the stress system. UCN3 is strongly expressed in both, neuronal cell bodies as well as fibers in all three nuclei, the VNLL, the INLL, and the DNLL (Figure 3B). UCN3 expression in VNLL seems to colabel with VGluT1, but less so in the INLL and DNLL (Figure 3B). A group of UCN3-positive neurons medial from the DNLL most likely belong to the brachium (Figures 3A,B).

Despite the strong UCN3 expression, the NLLs seem to be devoid of CRFR2 expression. Only very few CRFR2-positive fibers pass through the NLLs (Figure 3C). Instead, CRFR2 is strongly expressed in the paraleminscal nucleus (PL) located medial to the INLL (Figure 3C). The PL is not a principal auditory nucleus, but it receives auditory input and is potentially involved in audio-vocal feedback (Covey, 1993; Metzner, 1993; Feliciano et al., 1995; Hage et al., 2006; Varga et al., 2008). CRFR2-positive PL neurons are embedded in a dense network of VGluT1-positive fibers (Figures 3D,E). Almost all of the UCN3-positive PL neurons are also expressing calbindin (Figures 3F,G).

### Inferior Colliculus

**FIGURE 4:**
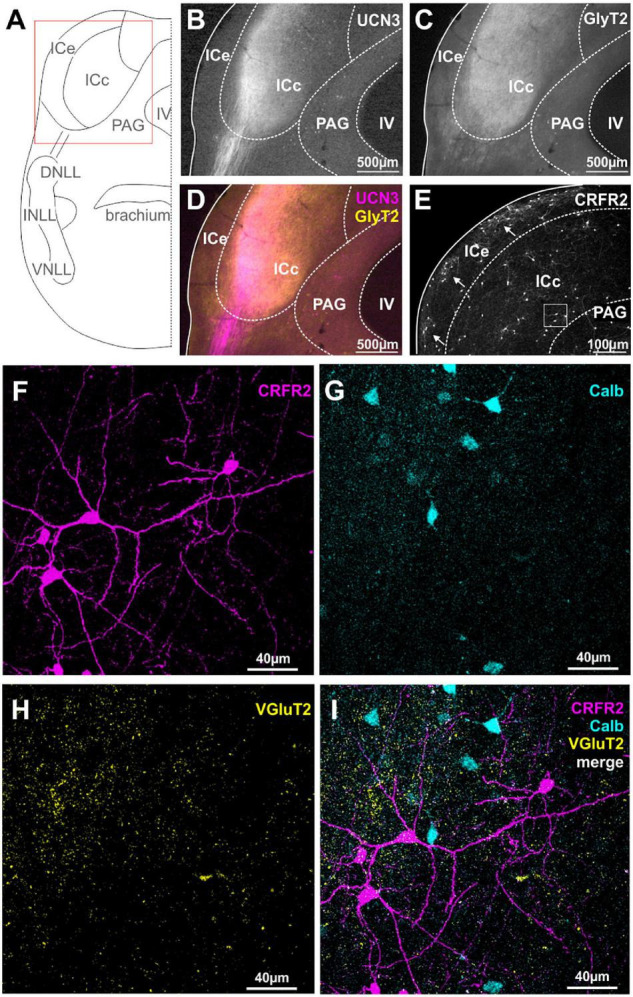
Spatial segregation of UCN3 and CRFR2 expression in the IC. **(A)** Schematic coronal section showing the inferior colliculus (IC) as the region of interest. IV, fourth ventricle; PAG, periaqueductal gray; NLLs, nuclei of the lateral lemniscus. **(B)** UCN3-positive fibers innervate the lateral part of the central nucleus of the IC (ICc). **(C)** GlyT2 expression in the IC. **(D)** Co-staining of UCN3 (magenta) and GlyT2 (yellow) shows a change in color of UCN3-positive fibers being magenta before entering the IC and appearing more whitish within the IC. UCN3 expression was not observed in the external (ICe) and dorsal (ICd) cortex of the IC. **(E)** Clusters of CRFR2-positive neuronal cell bodies in the ICe (white arrows). CRFR2-positive cells in ICc (white square) will be shown in higher magnification in **(F–I)**. **(F)** Higher magnification of CRFR2-positive cell bodies in ICc. **(G)** Calbindin-positive neurons in ICc. **(H)** VGluT2 labeling in ICc. **(I)** Merged image reveals no overlap between CRFR2 (magenta) and calbindin (turquoise) expression.

The IC is an auditory midbrain structure that receives ascending input from nearly all auditory brainstem nuclei, processes this information into new coding strategies and passes it on to the auditory thalamus. The IC is divided into the central core region (ICc) which harbors principal auditory neurons and into the external shell or cortex region (ICe) which receives multimodal inputs (Winer and Schreiner, 2005). Here, we report an extensive innervation of the ICc by UCN3-positive fibers (Figures 4A,B,D). More specifically, a long-range projection originating from the ipsilateral lateral lemniscus innervates the most lateral and ventral locations in the ICc. These again, as in case of the lateral calyces in the MNTB and the inputs to the lateral limb of the LSO, are areas containing low sound frequency-tuned cells. Strong GlyT2 labeling in the UCN3-positive areas of the ICc (Figure 4C), suggest that the UCN3-positive fibers may originate from the glycinergic VNLL neurons, rather than from the GABAergic DNLL neurons. Because GlyT2 antibodies only label the vesicular transporters in the glycinergic terminals but not along the axons, the UCN3-positive fibers appear purely magenta before entering the IC, but look more whitish upon entering the ICc, suggesting co-labeling with GlyT2 (Figures 4C,D). In contrast to the abundance of UCN3-positive fibers, neuronal cells bodies expressing UCN3 were only rarely observed in the ICc and in the dorsal IC (ICd; Figure 4B).

Similar to our observations on the DCN, the expression patterns of CRFR2 seems to be spatially segregated from the UCN3 expression. CRFR2 is predominantly expressed in cell bodies of ICe and to a much lesser extent in neuronal cell bodies of the ICd and ICc. Interestingly, within the ICe, CRFR2 expression is clustered in circular patches of tissue (Figure 4E) that have been previously described as expressing GAD67 and being the targets of somatosensory and other multisensory inputs to this area (Lesicko et al., 2016). The ICe is known to be an integrative-modulatory area, and the sources of this modulation also include descending projections from principal neurons in higher auditory centers (Adams, 1980). These descending projections, however, tend to segregate neatly outside of the aforementioned patches (Lesicko et al., 2016).

In the ICc and the ICd only few neurons are CRFR2-positive. These are scattered throughout the two subdivisions do not follow a clear pattern of distribution. The CRFR2-positive ICc neurons are characterized by a stellate morphology without a strong cellular orientation axis (Figure 4F). This suggest that these neurons are of the non-flat/disc-shape type that span multiple isofrequency laminae (Meininger et al., 1986). These CRFR2-positive ICc neurons are embedded in a dense network of VGluT2-positive fibers (Figures 4F,H,I). The CRFR2-positive neurons are also distinct from those which express calbindin (Figures 4F,G,I). Both of these observations indicate that CRFR2-positive ICc neurons are different from those ICc neurons that receive the main auditory input mediating sound localization information from the lower brainstem (Takahashi et al., 1987).

The expression patterns of UCN3 and CRFR2 in the IC lack obvious synaptic contacts between the ligand- and the receptor-expressing neurons, strengthening the hypothesis of non-synaptic means of volume transmission in this system.

### Medial Geniculate Body

The MGB is the auditory part of the thalamus and is composed of three main subdivisions, dorsal (MGBd), ventral (MGBv), and MGBm (Anderson et al., 2009). These subdivisions give rise to two major information streams to the cortex: the lemniscal stream (through MGBv) conveying ascending auditory information from the IC to Au1 (Winer, 1992; Anderson and Linden, 2011), and the non-lemniscal (through MGBd and MGBm) stream, conveying multimodal, more context-dependent information to secondary auditory cortex areas (Winer and Schreiner, 2005; Anderson et al., 2009). In addition, there are other parageniculate nuclei: the suprageniculate thalamic nucleus (SG), the posterior intralaminar thalamic nucleus (PIN), and the posterior limitans thalamic nucleus, which is also known as pretectothalamic lamina (PTL) (Anderson and Linden, 2011; Marquez-Legorreta et al., 2016).

**FIGURE 5:**
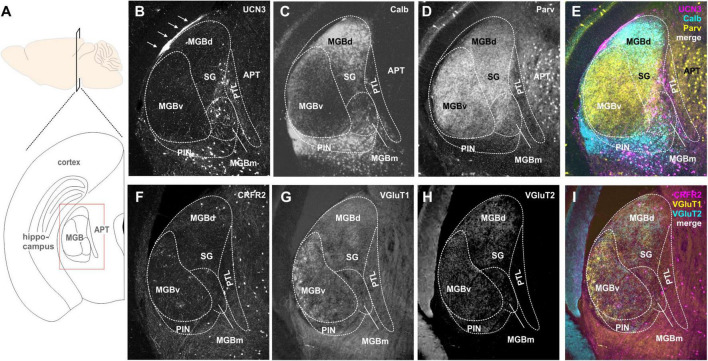
Expression of UCN3 and CRFR2 in the medial geniculate body (MGB) and the pretectal thalamic transition zones. **(A)** Sagital view of the brain indicating the level of the schematic coronal section (below) showing the medial geniculate body (MGB) as the region of interest. APT. anterior pretectal nucleus. **(B)** UCN3-expression in the medial subdivision of the MGB (MGBm), in the posterior intralaminar thalamic nucleus (PIN), and in the pretectal thalamic lamina (PTL). The thin layer of UCN3-positive fibers might occur in the marginal zone of the medial geniculate (white arrows). Calbindin **(C)** and parvalbumin **(D)** expression in the MGB were used as counterstain to identify substructures of the MGB. **(E)** Merge shows parvalbumin (yellow) expression in ventral MGB (MGBv), the suprageniculate thalamic nucleus (SG), and APT. Calbindin is mainly expressed in dorsal MGB (MGBd) and PIN. Neither parvalbumin nor calbindin show strong expression in the UCN3-positive neurons in MGBm and PIN. **(F)** Few CRFR2-positive neuronal cell bodies are present in the MGBm and the PTL. A network of CRFR2-positive fibers seems to surround the MGB without entering its core. **(G–I)** VGlutT1 **(G)** and VGluT2 **(H)** were used as counterstain.

Many UCN3-positive neuronal cell bodies were observed in the non-principal auditory areas like the MGBm, PIN, and PTL (Figures 5A,B). In addition, there are UCN3-positive fibers in the MGBm as well as a thin layer, which might be the marginal zone of the medial geniculate (Figures 5A,B). Counterstaining with parvalbumin, which in the brainstem is generally considered as an indicator for principal auditory neurons, showed only little overlap with UCN3-positive neurons in the MGBm (Figures 5B,D,E). However, in the MGB, many of the neurons in the MGBd and PIN are calbindin positive (Figures 5B,C,E), even though these areas are not considered to contain auditory principal neurons (Cruikshank et al., 2001; Lu et al., 2009; Marquez-Legorreta et al., 2016). This suggests that calbindin and parvalbumin might characterize different neurons in the thalamus compared to auditory brainstem and midbrain.

CRFR2-expression in the MGB was observed mostly in fibers and terminals (Figures 5F–I). These seemed to extent over several areas, but were especially prominent in the MGBm, PIN, and PTL. The origins of these CRFR2-positive fibers are most likely the multimodal domains of the IC, the ICe, and ICd. Additionally, a few CRFR2-positive neuronal cell bodies were present in the area of the PTL. However, even though the PTL also contained UCN3-positive neurons, at this point it is unclear if UCN3 and CRFR2 are expressed in the same cells. The lack of strong CRFR2 expression suggests that the receptor is expressed at a distant location like the amygdala where the UCN3-positive MGBm neurons project to. This would complete the pathway for auditory fear conditioning, hence providing an interesting link between auditory and stress system (Linke et al., 2000).

## Discussion

In this study, we characterized the expression patterns of the stress peptide UCN3 and its receptor CRFR2 in the mouse auditory pathway and found a strong presence in most subcortical structures. The combination of ligand and receptor expression allowed forming hypotheses about possible signaling mechanisms, which can be tested in future physiological experiments. In most auditory areas, a spatial segregation between UCN3-expression in auditory structures containing auditory principal neurons and CRFR2-expression in multisensory areas was observed (Table 1). This study introduces stress peptides as potential modulators of central auditory function.

**TABLE 1:** Distribution of UCN3- and CRFR2-positive neuronal cell bodies, fibers, and terminals in the auditory pathway.

**Brain area**	**Region/cell type**	**UCN3**		**CRFR2**		**References**
		**Cells**	**Fibers**	**Cells**	**Fibers**	
Cochlea	Inner hair cells (IHC)	−	+	−	+	Fischl et al., 2019
	Outer hair cells (OHC)	−	−	−	−	Basappa et al., 2012; Fischl et al., 2019
	Supporting cells			++	−	Basappa et al., 2012
	Spiral ganglion neurons (SGN)			+++	+	Graham et al., 2010
Cochlear nucleus	Bushy cells of the anteroventral cochlear nucleus (AVCN)	+	+			Fischl et al., 2019, This study
	Stellate cells of the anteroventral cochlear nucleus (AVCN)	++	+			This study
	Octopus cells of the posteroventral cochlear nucleus (PVCN)	−	−			This study
	Deep layers of the dorsal cochlear nucleus (DCN)	−	+++	−	−	This study
	Granule cell domain (GCD)	−	−	+++	+++	This study
	Small cells	−	−	++	−	This study
Superior olivary complex	MNTB	−	++	−	+	Fischl et al., 2019, This study
	VNTB	+	+	−	−	Fischl et al., 2019, This study
	SPN	++	+	−	+	Lewis et al., 2001; Li et al., 2002; Deussing et al., 2010; Fischl et al., 2019, This study
	LSO	+	+	−	+	Fischl et al., 2019, This study
	DPO	+++	+++	−	+	Fischl et al., 2019, This study
Nuclei of the lateral lemniscus	VNLL	++	++	−	−	This study
	INLL	++	++	−	−	This study
	DNLL	++	++	−	−	This study
	PL	−	−	++	−	This study
Inferior colliculus	ICc	−	+++	+	−	This study
	ICe	−	−	++	−	This study
	ICd	−	−	+	−	This study
Medial geniculate body	MGBv	−	−	−	−	This study
	MGBm	++	++	+	+	This study
	MGBd	−	−	−	−	This study
	SG	−	+	+	−	This study
	PIN	++	++	−	+	This study
	PTL	++	++	−	+	This study
	MZMG	−	+	−	+	This study

*The number of plus signs symbolizes the relative strength and a minus sign the lack of expression (Lewis et al., 2001; Li et al., 2002; Deussing et al., 2010; Graham et al., 2010; Basappa et al., 2012; Fischl et al., 2019).*

### Benefits and Shortcomings of Using Reporter Mouse Lines to Study Protein Expression

The expression of the fluorescent protein starts whenever the gene of interest turns on. It then produces the fluorescent protein, which will stay in the neurons. Therefore, the most common criticism of reporter mouse lines is the question of when in the lifetime of the animal the protein of interest is expressed. Consequently, UCN3 might not be expressed constitutively in all the auditory nuclei that we described in this study and it is possible that some of these expression patterns are developmentally regulated or are subject to specific stressful events. Nevertheless, utilizing these reporter models allowed us to observe the entire expression patterns that could occur for example in different behaviorally relevant situations or at different time points in an animal’s life. Since the influence of systemic stressors on auditory processing is not yet understood, knowing the auditory areas or cell types that can potentially be modulated by stress signaling is an important first step.

A major advantage of using reporter mouse lines for stress peptide signaling is that the expression can be visualized even when the target protein is very small and is expressed at very low concentrations. This aspect is crucial because neuropeptides are notoriously produced in very small quantities, mainly because they bind to G-protein coupled receptors (GPCRs), which have nanomolar sensitivities. This is in stark contrast to most ionotropic receptors, which have micromolar sensitivities (van den Pol, 2012). The small quantity and the small size of the peptide itself (38 amino acids for UCN3) makes detection with conventional immunohistochemistry particularly challenging. In addition, CRFR2 has some quite unusual characteristics that make it also difficult to detect *via* antibody binding. Specifically, CRFR2 contains a non-cleavable pseudosignaling peptide (PSP) attached to its N-terminal (Schulz et al., 2010; Teichmann et al., 2012). The PSP is absent from the other receptor type, CRFR1, and it confers some unique physiological traits of CRFR2 expression. In fact, PSP has been shown to anchor CRFR2 to the endoplasmatic reticulum and prevent its expression at the level of the membrane. This could potentially also impede the detection through receptor-mediated autoradiography, because the receptor might be inaccessible for radio ligand binding if the proper physiological triggers for membrane expression have not taken place. Tethering of the receptor intracellularly might also mask antigens for immunohistochemistry and hence much information could be lost by this technique. Besides, binding of the radiolabeled ligand has to compete with the endogenous ligand binding which might already be occupying the site.

Fluorescent *in situ* hybridization (FISH) and other genetic-based methods are also inferior to the use of reporter mice, because they can provide only a snapshot in time of the target’s expression that portrays only the genetic material actively being translated and therefore is highly dependent on any stress-related conditions of the animal prior to the sacrifice. Initial reports utilized FISH to study the general expression patterns of UCN3 and CRFR2 in the mammalian brain included information on the auditory system (Lewis et al., 2001; Li et al., 2002).

### Receptor-Ligand Mismatch

For decades, neuroscientists have reported a rather unexpected phenomenon regarding neurotransmitters and, even more so, neuropeptides: an apparent lack of direct synaptic contact between neurons expressing a receptor and those expressing its high-affinity ligand in certain brain areas and cell types (Herkenham, 1987). An observation that seems to contradict the dogma of synaptic transmission. Even if acknowledged, this phenomenon has not spurred extensive investigation, with a few notable exceptions (Agnati et al., 1995; Ludwig and Leng, 2006). Very often, this intriguing finding has been attributed to technical limitations as explained in the previous paragraph. However, other possible solutions to this mystery have been explored and even proved for some neuropeptides (Liu et al., 1994; Liu and Sandkuhler, 1995; Brown et al., 2008; Ha et al., 2013). First of all, one has to consider that neuropeptides, in contrast to classical neurotransmitters, could reach their target receptor over very long distances through blood circulation. That is the case for example for leptin, ghrelin, and insulin, which are released at the level of the gastrointestinal system, but also affect the central nervous system (Rhea et al., 2018; Stengel and Tache, 2018). Not all neuropeptides can cross the blood–brain barrier; however, experimental evidence shows generalized effects of UCN3 after central injections, supporting a neuroendocrine option for UCN3 (Sharpe and Phillips, 2009; Yeh et al., 2016).

Besides the long-range endocrine signaling, some neuropeptides such as galanin (Freund-Mercier and Stoeckel, 1995) and oxytocin can also be released by dendrites and neuronal somata (Vila-Porcile et al., 2009). In invertebrates, where mechanisms of neuropeptide release have been studied more intensely, it has been shown that neuropeptides are shuffled anterogradely and retrogradely within the same neuron using different molecular motors (Barkus et al., 2008; Wong et al., 2012).

Finally, the lack of synaptic re-uptake mechanisms and the presence of extracellular peptidases are strong indicators that volume transmission plays a major role in many instances. However, due to the presence of the extracellular peptidases, most of the effects remain local and limited in time (Defea et al., 2000). This is emphasized by the fact that neuropeptides and their receptors have an overall discrete expression in certain areas. A more widespread diffusion would defeat the purpose of this specialization.

### Possible Binding of CRFR2 by Other Ligands

The possibility, that in some areas UCN3 might not be the only ligand for CRFR2 has to be taken into account when making functional considerations. UCN2 can also bind to CRFR2 with high affinity, UCN1 can bind with moderate and CRF with low affinity. To date, there is no report about the presence of UCN2 in the auditory system. However, CRF positive neuronal cell bodies are present in the DCN, lateral MTNB, ICe, DNLL, VNLL, and MGB (Imaki et al., 1991). Although CRF has a very low affinity for CRFR2 and would have to be released at extremely high concentrations to sufficiently occupy the receptor, it cannot be excluded that CRF might also act on CRFR2 receptors in the auditory pathway. Despite possible binding of CRFR2 by UCN2 or CRF, UCN3 is still the most likely ligand (Li et al., 2002). In contrast to the other ligands, UCN3 binds exclusively to CRFR2, and so its sole purpose is to be released and to bind this receptor. Hence, the spatial segregation between presynaptic UCN3 and postsynaptic CRFR2 expression that we observed in multiple auditory areas, could not simply be explained by postulating that in these areas CRF rather than UCN3 might be the main ligand. Moreover, it suggests that if UCN3 is expressed it has to bind to its exclusive receptor CRFR2 to have a functional effect even if it involves volume transmission.

Volume transmission might be corroborated by the fact that another ligand-receptor pairs of stress peptides also shows a segregation of ligand and receptor. In the IC, the ligand CRF is expressed in the integrative cells of the external cortex (Imaki et al., 1991), whereas its receptor, CRFR1 is expressed in principal auditory neurons of the central nucleus (Justice et al., 2008). A note of caution has to be given though, that the latter publication was not specifically focused on the auditory system and the identity of the cell types expressing CRFR1 was not clearly established.

Nevertheless, the general presence of other ligands gives an interesting perspective on how this system could be extremely well refined for balanced modulation. For example, certain stressors might be too mild to release a large enough quantity of CRF to compete with UCN3 for CRFR2 binding, which would make the modulation modality- and/or intensity-dependent. Alternatively, CRF might be released by a different population of neurons than UCN3, which is mostly released by neurons tuned to low sound frequencies.

### Functional Implications of Urocortin 3 and CRFR2 Expression in the Auditory System

The most intriguing finding of our study is that the neuromodulatory ligand UCN3 tends to be expressed in auditory principal neurons, whereas CRFR2 labeling is mostly found in non-principal, multimodal neurons. Such a distribution is unusual, because typically the auditory principal neurons are the target of neuromodulatory inputs rather than being their source (Schofield et al., 2011; Sizemore et al., 2020). An obvious example is the CN where UCN3 is expressed in the magnocellular core of both VCN and DCN while CRFR2 is mostly found in the granular cell domain and the parallel fibers. Similarly, in the IC, UCN3 positive fibers are found within the central nucleus, while CRFR2 is expressed in the external cortex of the IC.

Normally, we would consider modulation as coming from other systems, that are either cross-modal or are devoted to modulation itself like the reticular formation, both of which would then alter the incoming auditory information. Indeed, the presence and function of modulatory inputs to auditory structures have been described in many studies (Ryugo et al., 2003). For example, a large body of work on the DCN revealed how somatosensory inputs from head and neck can suppress self-generated sound perception (Shore, 2005; Koehler and Shore, 2013; Singla et al., 2017). A lot of clarification came from identifying the anatomical origin of these modulatory fibers and in-depth mechanistic explanations (Trussell and Oertel, 2018). More so, clinical evidence from the treatment of certain types of tinnitus utilizing cranio-cervical manipulations provided additional strength to these data (Levine, 1999).

However, the occurrence of reverse patterns of modulation as we describe here for the UCN3–CRFR2 system is novel and its functional significance is up for discovery. Although a few cross-modal feedback projections from auditory structures might form part of the UCN3–CRFR2 system, to our knowledge, the finding of an on-site modulation of stressful situations has not yet been explored in the central auditory pathway.

The interesting scenario here is that sound-driven release of the ligand might affect the cells responsible for receiving external modulation and set them up for specific types of firing. For instance, release of UCN3 from fibers reaching the central nucleus of the IC might bypass the principal auditory cells in ICc and instead directly modulate the activity of the multimodal cells in the ICe *via* volume transmission. The timescale of volume transmission is certainly an interesting aspect with regard to the fast signal processing of primary ascending auditory information. Non-gaseous neuromodulators including UCN3 are long-lived and their lifetime is typically determined by the tissue-specific degradation processes (for review see, Russo, 2017). Although, to our knowledge, the lifetime of UCN3 in the brain is not yet known, the lifetime of other neuropeptides such as oxytocin and vasopressin in the cerebrospinal fluid is reported to be up to 20 min. These 20 min together with the downstream G-protein coupled signaling cascade of UCN3 and most other neuropeptides suggest the action of UCN3 to be slow compared to the primary auditory signal processing. However, since processing of auditory information takes place at different time scales from microseconds to many seconds or even hours, UCN3 signaling is just in time to modulate processes involved in temporal abstraction rather than temporal resolution or temporal integration (Kopp-Scheinpflug and Linden, 2021). Such a setting could maintain faithfulness of direct ascending auditory transmission on one hand, while at the same time interfering with the modulatory effects of the ICe cells and their inputs to non-auditory areas.

Another interesting finding is that CRFR2 expression in the SOC seems less abundant than that of UCN3. Instead, CRFR2 expression in surrounding non-auditory areas of the cranial nerve and nuclei of the reticular formation is very strong, suggesting that one purpose of UCN3-release by SOC neurons might be to affect surrounding non-auditory structures rather than afferent auditory principal neurons. With respect to the SOC as an evolutionary highly conserved collection of nuclei that are essential for the execution of precise and survival-promoting encoding of sound information, too much modulatory impact could even be detrimental in the SOC. An exception of the UCN3 positive structures in the SOC are the calyces of Held innervating lateral, low-frequency tuned MNTB neurons. Here, we found an area in which an autocrine modulation could take place, since these lateral calyces of Held express both the receptor and the ligand. It is still unknown, whether the same calyces express both ligand and receptor; a question that will have to be answered by single cell physiology or through the generation of a double reporter mouse line. In this case, the autocrine route seems to be the prevalent one, even if it does not exclude that volume transmission is also happening. In fact, it is known that neuropeptides can travel up to hundreds of micrometers to reach their receptor (Fuxe et al., 2010); yet the concentration that actually reaches the furthest targets declines linearly with the amount to extracellular peptidases being expressed along the way. To date, information on the presence and localization of extracellular peptidases is still lacking in these auditory areas. Hence, it seems most reasonable that the CRFR2 expressing calyces in the lateral MNTB would receive the majority of UCN3 released within the same area compared to other CRFR2 expressing cells more at a distance. Similar possible combinations of autocrine and paracrine signaling has been suggested for CRF-CRFR1 signaling between cochlear supporting cells (Graham et al., 2011).

The strong expression of UCN3 in VNTB neurons is most likely due to its contribution in the efferent feedback system of the medial olivocochlear complex (MOC). MOC neurons project to the outer hair cells of the cochlea and protect these during damaging sound intensities. However, prior research on the contribution of UCN3 during sound over exposure did not show an effect on cochlear outer hair cells (Fischl et al., 2019). Efferent projections of MOC neurons also send collaterals into the CN targeting the small cell cap. Here, we showed that the small cells express CRFR2 and therefore qualify as the MOC-UCN3-target. Indeed, a very recent study investigating the physiology of CN small cells suggest a special role for these cells in processing communication sounds, a function that is most certainly subject to stress and emotional modulation (Hockley et al., 2021).

## Conclusion

With this work, we aim to highlight the presence of the UCN3–CRFR2 system as so far unexplored neuromodulators in the central auditory pathway. First, the expression of both factors is widespread in subcortical auditory nuclei. Second, the ligand-receptor expression patterns suggest unusual forms of neurotransmission such as local or long distance volume transmission and possibly even autocrine regulation. Third, an interesting pattern of segregation between the ligand being expressed in auditory principal cells and the receptor being expressed in non-principal neurons implies a stress-dependent modulation of the canonical modulators. These results open up a new field of research, investigating which stressors could be activated under what circumstances and how these stressors influence central auditory processing.

## Data Availability Statement

The raw data supporting the conclusions of this article will be made available by the authors, without undue reservation.

## Ethics Statement

The animal study was reviewed and approved by the Bavarian district government.

## Author Contributions

All authors contributed to the conception and design of the study and data collection. SP performed experiments and wrote the first draft of the manuscript. All authors contributed to manuscript revision, and read and approved the submitted version.

## Conflict of Interest

The authors declare that the research was conducted in the absence of any commercial or financial relationships that could be construed as a potential conflict of interest.

## Publisher’s Note

All claims expressed in this article are solely those of the authors and do not necessarily represent those of their affiliated organizations, or those of the publisher, the editors and the reviewers. Any product that may be evaluated in this article, or claim that may be made by its manufacturer, is not guaranteed or endorsed by the publisher.
